# Effect of Wheat Bran Incorporation on the Physical and Sensory Properties of a South African Cereal Fried Dough

**DOI:** 10.3390/foods8110559

**Published:** 2019-11-07

**Authors:** Fortunate N. Ndlala, Oluwatoyin O. Onipe, Tabea M. Mokhele, Tonna A. Anyasi, Afam I. O. Jideani

**Affiliations:** Department of Food Science and Technology, School of Agriculture, University of Venda, Private Bag X5050, Thohoyandou 0950, Limpopo Province, South Africa; ndlalafortu@gmail.com (F.N.N.); toyin.onipe@gmail.com (O.O.O.); tabea.mokhele@univen.ac.za (T.M.M.); tonna.anyasi@gmail.com (T.A.A.)

**Keywords:** cereal fried dough, wheat bran, texture profile, sensory profile, consumer acceptability

## Abstract

This study investigated the effect of wheat bran (WB) supplementation on the physical and sensory properties of a South African cereal fried dough (*magwinya*). The physical properties, instrumental texture, and sensory profile were determined for *magwinya* (100:0, control) and for wheat flour to wheat-bran ratios of 95:5 (MWB5), 90:10 (MWB10), 85:15 (MWB15), and 80:20 (MWB20). An increase in the proportion of WB in the fried dough showed no significant difference on the specific volume (1.47–1.54) of samples. The chroma value (30.19–22.29), lightness (35.92–28.98), and hue angle (55.03–47.77) decreased, while ∆E increased distinctly with the addition of WB. *Magwinya* supplemented with WB was less cohesive and easy to chew. Significant correlations were found between instrumental hardness and sensory springiness (*r* = −0.63; *p* < 0.05), as well as between instrumental cohesiveness and sensory springiness (*r* = −0.71; *p* < 0.01). Two principal components were identified, which accounted for 85.1% of the variance in the instrumental data. A substitution level of 5 and 10% WB was similar to the sensory properties of the control in taste, texture, and overall acceptability and can replace part of the wheat flour in the cereal fried dough production.

## 1. Introduction

*Magwinya*, a prevalent street snack in South Africa, is a deep-fried dough which can take up a large amount of oil during the frying process. The snack is a fermented confectionary produced from wheat flour, sugar, yeast, salt, and lukewarm water by processing operations such as mixing, kneading, shaping, and frying. It is a golden-brown fried snack, round, crisp crust, and has an innermost part like a baked rather than a fried product. Although not yet categorized in the healthy snack group, *magwinya* is quite popular [1]. 

Bran is made up of the hard-outer layers of cereal grain such as corn, wheat, rice, oats, barley, rye, and millet [2]. This outer layer provides fiber, minerals, B vitamins, and bioactive compounds that increase bulk and regulates the absorption and excretion of nutrients from the body [3]. Wheat (*Triticum aestivum*) is one of the massively produced cereals mainly used for human consumption and livestock feed. The human consumption of wheat bran (WB) has gradually increased over the years, as shown by a 48% worldwide increase in wheat bran-incorporated food products over a ten-year period [4]. The incorporation of WB in fried foods leads to the formation of a barrier that limits the movement of fat. This is due to the fact that WB has been reported to possess a potential for fat reduction in fried products [4]. WB is utilized as an additional source of dietary fiber and helps in the relief of constipation by speeding up the intestinal transit time, increasing stool output, and bowel movement frequency, thus preventing colon cancer. Regular consumption of dietary bran-rich foods can also reduce the danger of hypercholesterolemia, hypertension, breast cancer, and type 2-diabetes. Similarly, the addition of WB in cereal-based products helps certain rheological changes such as increased dough, water absorption, and reduced loaf volume, thereby enhancing the quality characteristics of end products [5]. Food produce enriched with WB includes bread, muffin, bran flakes, yogurt, *tarhana* soup, doughnuts, pasta, noodles, and biscuits.

Sensory evaluation is defined by Lawless & Heymann [6] as a scientific method for measuring, analyzing and interpreting human responses to products as perceived through their sense of touch, taste, sight, smell, or sound. It is always used in assessing consumer’s responses during the development of existing or new products. Texture, a sensory attribute, is a presentation of the food’s structure and response to force [7]. The classification of texture attributes into categories introduced by Szczesniak et al. [8] gave rise to a profiling method of texture description. Such a texture profile analysis (TPA) is applicable to both sensory and instrumental measurements. Texture profiling with instrumental method involves test substance compression and quantifying the mechanical parameters from the force-deformation curves recorded [9]. There, however, exist a lack of information on the instrumental texture and sensory profile of *magwinya*. This research thus seeks to fill that gap with the information that will be generated.

## 2. Materials and Methods

### 2.1. Samples and Sample Preparation

Coarse wheat bran (Snowflake, South Africa), cake wheat flour (Pioneer Foods, South Africa), instant dry yeast (Rymco Ltd., South Africa), sugar (“Selati” TSB Sugar, Malelane, South Africa), salt (Cerebos, South Africa), sunflower oil (Spar, South Africa) were purchased at Shoprite and Spar grocery stores, Thohoyandou, South Africa. Five sample blends used for wheat bran *magwinya* production were prepared based on wheat flour to wheat-bran ratios of 100:0 (control sample), 95:5 (MWB5), 90:10 (MWB10), 85:15 (MWB15) and 80:20 (MWB20).

**Figure 1:**
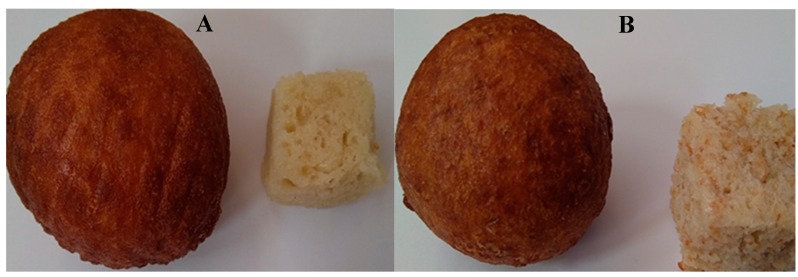

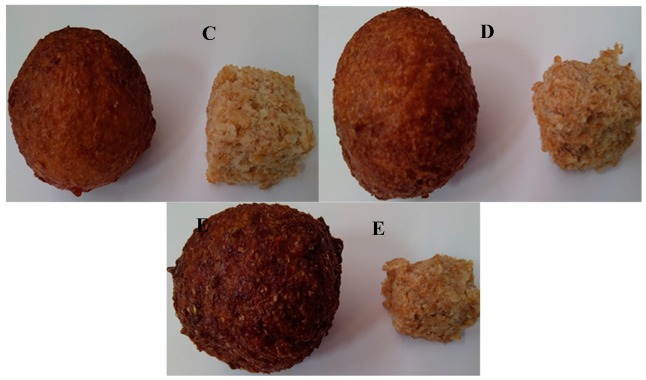
South African cereal fried dough (*magwinya*) samples (left: whole sample; right: crumb). **A** = 100% WF (control); **B** = 95% WF + 5% WB; **C** = 90% WF + 10% WB; **D** = 85% WF + 15% WB; **E** = 80% WF + 20% WB. WF = wheat flour; WB = wheat bran.

The traditional method of Kwinda et al. [10] was employed in the process of *magwinya* (control) and wheat bran *magwinya* production. The following ingredients: composite flour (100 g), salt (1 g), sugar (15 g), yeast (1 g), and 100 mL lukewarm water (25–26 °C) were weighed and mixed manually for 5 min to obtain a homogenous wet sticky dough which was formed before fermentation at ambient temperature 21 °C for 45 min. The dough was squeezed into cooking oil and fried for 5 min at 180 °C using a fryer (Russell Hobbs, model RDF 300, UK) to produce the different composite cereal fried dough samples (Figure 1).

### 2.2. Volume and Weight Measurement of Wheat Bran Magwinya Samples

The rapeseed displacement method of the American Association of Cereal Chemists (AACC) [11], method number 10-05-01, with some modifications, was used for the determination of sample volume. A 1000 mL beaker was filled with finger millet grains. The grains were then poured out and used in refilling the box containing wheat bran *magwinya* samples. The final level of the grain minus the initial level obtained was taken. The volume of displaced grain is equal to the volume of the wheat bran *magwinya* samples. The final weight of the cooled cereal fried dough samples was measured using a weight balance. 

### 2.3. Specific Volume of Wheat Bran Magwinya Samples

The specific volume was obtained by dividing the loaf volume of wheat bran fried dough by its corresponding weight, as shown in Equation (1) [12].

(1) $$\mathrm{Specific}\;\mathrm{volume}\;=\;\frac{V\;\left({\mathrm{cm}}^{3}\right)}{\mathrm{Wt}\;\left(g\right)}$$

### 2.4. Color Determination of Wheat Bran Magwinya Samples

Color analysis was performed following the method of Onipe et al. [13] in a ColorFlex 45/0 Spectrophotometer (Hunterlab, Reston, VA, USA) with illuminant D65 and a 10° observer. The instrument was calibrated by covering a zero-calibration mask (CM-A124) followed by the white calibration plate (CM-A120). The samples were then analyzed by placing them on the sample holder (CM-A128) covered with a black container. Three replicates of the crust and crumb for each wheat bran *magwinya* sample were analyzed for L* (luminosity), a* (opposition of colors green and red), and b* (opposition of colors blue and yellow). Chroma (C*), hue (h), and total color difference (∆E) were calculated using Equations (2)–(4).

(2) $$\mathrm{Chroma}\;=\;\sqrt{a^{2}+b^{2}}$$

(3) Hue= tan-1(ba)

(4) $$\Delta E\;=\;\sqrt{\left[{\left(L\;-\;\mathrm{Lc}\right)}^{2}\;+\;{\left(a\;-\;\mathrm{ac}\right)}^{2}\;+\;{\left(b\;-\;\mathrm{bc}\right)}^{2}\right]}$$

where Lc, ac, and bc represent color values for the control sample.

### 2.5. Texture Profile Analysis

The standard method of AACC [11] method 74-09 with slight modifications was adopted in the measurement of texture profile (hardness, springiness, cohesiveness, and chewiness) of composite cereal fried dough samples using a texture analyzer TA-XT2i (Stable Micro Systems, Godalming, England). Fresh samples were used for analysis after cooling to ambient temperature for 1 h. Before conducting the test, the probe was calibrated. Samples with sides of about 2 cm were cut from the wheat bran *magwinya* crumb and placed centrally beneath the probe ((p/36 R cylinder probe (36 mm)) to meet with a consistent flat surface. The compression test was selected in the texture analysis using a 5-kg load cell, and the sample was compressed to 35% of its original height. A two-cycle compression was set with a strain force of 50%, pre-test speed: 1.0 mm, test speed: 2.0 mm, post speed: 2.0 mm, and trigger force of 5 g. Readings were carried out in triplicates and sensory attribute parameters obtained from the curves. The four TPA parameters of hardness, cohesiveness, springiness, and chewiness were calculated at the time of the test by determining the load and displacement at predetermined points on the TPA curve. Hardness was obtained as the maximum load expressed in kg and applied to the samples during the first compression. Cohesiveness was obtained as the ratio of the area under the curve for the second compression to that under the curve for the first compression. Springiness was expressed as the ratio of the duration of contact with the sample during the second compression to that during the first compression, while chewiness was expressed as the mathematical product of hardness, cohesiveness and springiness. 

### 2.6. Descriptive Sensory Analysis

**Figure 2:**
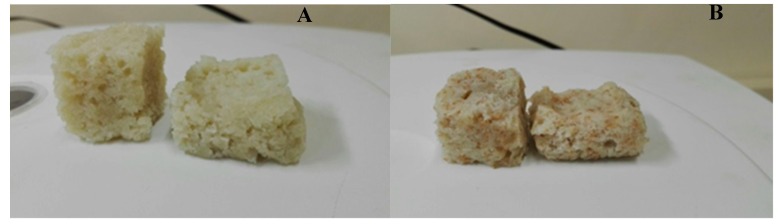
Illustrations showing texture analysis of *magwinya* samples. **A** = 100% WF (control); **B** = 95% WF + 5% WB.

Eight-member trained sensory panel was used for the analysis. The members were selected using screening tests, which included taste (bitter, sweet, sour, salty, and umami) identification and exercises to describe differences among the composite cereal fried dough. The generic description analysis method [14] was used for determining the sensory profile of the wheat bran *magwinya* samples. The sensory panelists were trained in ten sessions of 2 h per day during which a total of twenty-one sensory attributes (appearance, aroma, texture, flavor, and aftertaste) with definitions, reference standards and methodology of the evaluation were developed (Table 1). *Magwinya* samples were cooled for 1 h on medium-sized stainless steel cooling trays before slicing to a uniform thickness and placed on a transparent plastic container coded with random three-digit codes. Each attribute was evaluated on a 10-point scale (1–10) anchored with verbal descriptions. Evaluation of the five wheat bran *magwinya* samples and control was replicated three times in sessions of 1.5 h per day for three days. This was done in a sensory laboratory with individual booths under white light at room temperature following standard good sensory practices (Figure 2). Training procedures for panelists followed the guidelines of Munoz [15] and Meilgaard et al. [16] with bottled water provided as palate cleansers and responses collected using questionnaires.

### 2.7. Consumer Acceptability Test

Evaluation properties of appearance (crust color, crumb color), texture, taste, aroma, flavor and overall acceptability of wheat bran *magwinya* samples were conducted as described by Meilgaard et al. [16]. Wheat bran cereal fried dough samples for the panelists’ evaluation were coded with three-digit numbers and presented randomly to eighty (80) untrained panel members that were recruited at the University of Venda, South Africa. The panelists comprising males and females of different age groups were given consent forms each to fill before being asked to analyze the samples based on the evaluation properties. A nine-point hedonic scale test having 9 as like extremely, 5 as neither like nor dislike, and 1 as dislike extremely was used in the evaluation of wheat bran *magwinya* quality. The samples were sliced into portions of about 50 g that included both the crust and crumb. The intention of creating a context in which all wheat bran *magwinya* were assessed was to focus all participants on the concept of likeness. Participants were then asked to observe, smell, and taste each sample of the composite cereal fried dough. After evaluating each sample, participants then judged sample likeliness using the questionnaires provided. Bottled water was provided as a palate cleanser, and a break of 60 s was taken before evaluating the next sample. 

### 2.8. Statistical Analysis

The mean and standard deviation (SD) of the analyzed data are the results of triplicate analysis. A one-way analysis of variance (ANOVA) at 95% confidence level (*p* ≤ 0.05) using Duncan’s Multiple Range Test was used in separating the means of triplicate determinations. Statistical analysis by ANOVA for determining the effect of level of bran addition (independent variables) on parameters measured (dependent variables) was done using IBM SPSS Statistics for Windows version 22 (IBM Corp., Armonk, NY, USA). Triplicate scores were further averaged, standardized (1/standard deviation), and subjected to principal component analysis (PCA) using Minitab version 17 (Minitab Inc, State College, PA, USA). 

## 3. Results and Discussion

### 3.1. Color Profile of Wheat Bran Magwinya

Color is one of the main factors that affect food product acceptability at the time of consumer purchase [17]. The color of the crust and crumb has been an important parameter for fried products. Fried products are likely to obtain a dark crust, and significant limitation of product darkening could negatively affect the sensory properties of the final product. The color (L*, a*, b*) characteristics of crumb and crust of wheat bran *magwinya* samples are presented in Table 2. Crumb of the cereal fried dough samples partially substituted with WB had significantly (*p* < 0.05) lower L* values compared to the control. Increasing the amount of WB in wheat bran *magwinya* formula, gradually reduced the L* with the significant difference among all-composite fried dough samples. Almeida et al. [18] also reported a decrease in luminosity in bread crumb with increased addition of wheat bran from 5–20%. 

The crumb and crust of *magwinya* samples containing WB were darker compared to the control. This observed trend was supported by decreasing L* values as the WB content increased in the cereal fried dough (Table 2). The crust of sample MWB20 was significantly darker than MWB15, MWB10, MWB5, and the control. This decrease in lightness can be majorly attributed to the influence of the dark color of WB introduced to the dough formulation. In addition, caramelization and Maillard reaction, which occurs during frying, caused the color change in the crust of wheat bran *magwinya* [13,19]. Crumb redness (a*) values significantly increased as WB content increased in the samples. Wheat bran has been reported to have high redness intensity [20]. Therefore, its incorporation in the cereal fried dough increased the redness of the crumb of the composite fried, though. Crumb yellowness (b*) values reported in this work were in close range to those reported by Onipe et al. [13]. This might be due to the brown pigment of WB. The yellowness of the crust of wheat bran *magwinya* was significantly different from the control. Chroma, as stated in the works of Pathare et al. [21], describes the intensity of colorfulness perceived by humans. Chroma values of the crumb (20.23–21.65) were lower than that of the crust (22.29–30.19). This shows that the intensity of color in the crust is much more than the crumb due to wheat bran incorporation. Wheat bran *magwinya* crumb had significantly higher (*p* < 0.05) Chroma values when compared to the control. Conversely, the crust of the cereal fried dough showed a reduction in the Chroma value upon the addition of WB. The crumb and crust hue angle values of the fried dough containing WB were significantly lower than the control samples (Table 2). Hue angle qualitatively describes the traditional color of a product in terms of actual colors like red or green. Hue angles of the crumb ranged from 65.46° to 84.55° while that of the crust was from 47.57° to 55.03°. The hue values fall between 0° and 90° for which 0° represents red, while 90° represents yellow [21].

### 3.2. Weight, Volume, and Specific Volume Measurement

The weight of wheat bran cereal fried dough samples ranged from 43.10 to 53.83 g (Table 3) with a significant difference (*p* < 0.05) observed among these values. The control sample-specific volume was not significantly different upon the addition of 5–20% WB. During *magwinya* sample production, the dough was squeezed using a hand to frying oil, which produced non-uniform *magwinya* samples as a result of inconsistent dough size. Kim et al. [22] reported in their work that loaf volume, as well as specific loaf volume, decreased significantly with an increased proportion of WB in bread. Samples MWB5 and MWB10 (which represent the lower portion of WB incorporation) had the highest volume. This is most likely due to the higher level of gluten present in wheat flour compared to composite blends, which could not properly retain carbon dioxide gas during proofing. An increased loaf weight might be due to the WB that absorbed high-water and the reduced air entrapment, resulting in heavy dough [23]. 

### 3.3. Textural Profile Analysis (TPA)

Textural properties play a remarkable role in the perception and acceptability of any processed food product. Processing conditions and ingredient formulations show a direct impact on the textural behavior of food products. The texture properties of *magwinya* were described using hardness, cohesiveness, springiness, and chewiness attributes (Table 4). Furthermore, while hardness relates to the force applied by the molar teeth to compress the food, chewiness relates to the number of chews necessary for food to be swallowed. Cohesiveness relates to the amount of deformation undergone by a material before rupture when biting completely through the samples using molars. Springiness relates to the degree or rate at which the sample returns to its original size/shape after partial compression between the tongue and palate [24,25]. The WB added to the cereal fried dough decreased cohesiveness and chewiness while an increase was observed for hardness. Regarding springiness, there was no observed change (*p* > 0.05) in the different concentrations of WB (Table 4). However, an increase in *magwinya* hardness with an increasing amount of WB occurred in all four cereal fried dough samples. *Magwinya* produced using WB-wheat flour composite exhibited harder texture than the control (100% wheat flour), although similar results were reported by Sobota et al. [26]. The hardness in baked food production is affected by factors such as moisture content and migration as well as the redistribution of water in a product. Other factors, such as gluten-starch interactions and starch retrogradation, are vital phenomena contributing to the hardness of fried or baked products [27]. As reported by Shittu et al. [28], starch after bread baking, retrogrades and gels within the inter-granular spaces, leading to rigidity and hardening of bread.

Suspension refers to the rate at which a deformed product returns to its undeformed state when the deforming force is removed, while chewability is the energy required to bring solid foods into a state of ready to swallow. Both phenomena are directly related to hardness and chewiness [29]. In general, a change in substitution level did not show a significant effect on elasticity and chewing. The increase of WB in the flour gave *magwinya* a less cohesive and easy to chew characteristic. The elasticity of cereal fried dough substituted with 5, 10, 15, and 20% WB was comparable with the control. Chewiness of *magwinya* from 15% WB compared well with that of 20% WB. Conversely, samples from 5 and 10% WB and control differed markedly from those of 15 and 20% WB, like those of 15 and 20% WB were observed to be the easiest to chew. As observed for hardness, elasticity was seen to be strongly influenced by moisture content, moisture redistribution, and retrogradation of the starch. Therefore, increasing the WB fraction is likely to result in a greater downgrade upon the cooling of fried *magwinya* [27].

### 3.4. Descriptive Sensory Analysis of Wheat Bran Magwinya

The descriptive sensory analysis gives good insight on nature and size (quantity or intensity) of sensory attributes as perceived by humans while consuming food products. The product profiling information gathered with the method assists in understanding the effect of ingredients on sensory properties of products useful for predicting potential consumer responses to new products [6]. Table 5 shows the effect of WB addition on *magwinya* sensory properties. The choice of cereal (wheat bran) significantly influenced the appearance, aroma, and flavor attributes of *magwinya* samples. Cereal fried dough incorporated with 20% WB received higher ratings compared with fried dough without WB (control) for appearance, aroma, aftertaste, and texture. Cohesive texture increased with the addition of WB, and this increase indicates that *magwinya* prepared with WB possess the higher ability of resistance towards *magwinya* structure deformed under the teeth. With the use of the descriptive sensory method, evaluation included several steps outside and inside the mouth: from the first bite through mastication, swallowing, and residual feeling in the mouth and throat. The application of this method is, however, based on standard scales [30]. 

The springiness of the cereal fried dough samples significantly (*p* < 0.05) increased upon the addition of WB in their formulation. The springiness of the fried dough samples containing 15 and 20% WB showed a significant difference (*p* < 0.05). As observed in the works of Hoseney et al. [31], the interaction between gelatinized starch and gluten dough causes the dough to be more elastic, thereby leading to the formation of a continuous sponge structure of bread after heating. Thus, the high springiness in MWB15 and MWB20 (Table 5) could be attributed to dilution of the gluten structure in composite *magwinya*. Sample MWB20 was observed to be hard and difficult to chew than the control, which might be due to the higher gluten content of the samples. The addition of WB gluten flour in the production of the cereal fried dough increased the gluten strength of the wheat flour, hence strengthening the overall structure of the fried dough. This observation agrees with the work of Sabianis and Tzia [32], who reported that the hardness of bread increased as the amount of WB increased. Similarly, the addition of WB increased ratings for crust brownness, dry aftertaste, evenness appearance, and toasted ‘burnt’ aroma, while ratings decreased for crumb lightness and crust smoothness. Other properties of grittiness, lingering, and dryness of *magwinya* incorporated with WB can be minimized by reducing the WB particle size from coarse to fine particle size through milling of the bran. As reported by Zhang and Moore [33], bread containing fine particle size bran had lighter crust color, better crust appearance, and less gritty mouth-feel than bread containing coarse or medium particle size bran.

### 3.5. Principal Component Analysis of Sensory and Instrumental Properties of Magwinya

**Figure 3:**
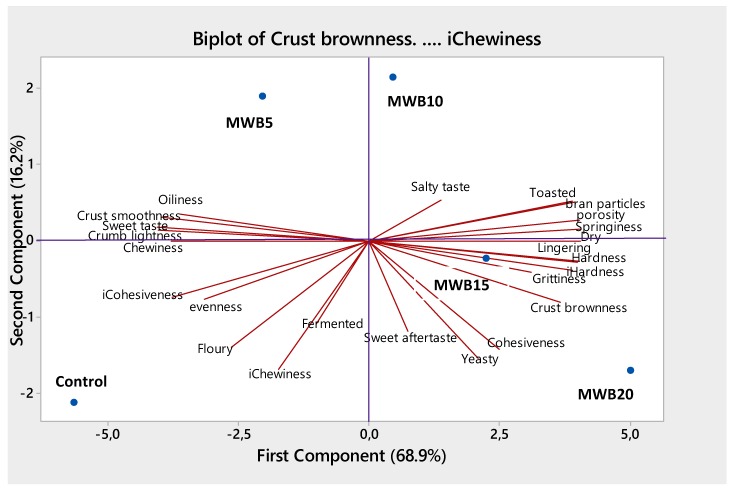
Principal component analysis plot of wheat bran cereal fried dough with loading coordinates for sensory properties.

Principal component analysis (PCA) on the sensory and instrumental data (Figure 3) showed that the first and second principal components represented 16.2% and 68.9% of the observed variation (85.1% in total). PC1 separated the cereal fried dough based on the addition of WB, with PC2 WB *magwinya* to the left and top right, while *magwinya* without WB more to the bottom left. Cereal fried dough with 10, 15, and 20% WB were identified as more aftertaste intense with a lingering, grittiness, and drier aftertaste. The lack of aftertaste, texture, and flavor attributes at the bottom left of the plot shows that the control *magwinya*, in contrast, was generally perceived as the norm. Visual and color attributes, crust brownness, and hard texture, are also distinctive factors of PC1. PC2 separated the fried dough with added WB at a different concentration at the top and bottom right, from the control towards the bottom left. Control *magwinya* had a floury and fermented aroma and a more even appearance. The aftertaste of *magwinya* with WB was observed to be more dry, lingering, and gritty, but the control fried dough had a more intense overall floury aroma. The more intense floury taste might be due to the volume of flour used without further addition of composite ingredients. In general, it was observed that cereal fried dough with WB was more cohesive, had a more dry, lingering and gritty aftertaste, hard texture, and toasted aroma compared to the control *magwinya*. Hardness is mainly attributed to the amylose and amylopectin matrix, which contributes to overall bread texture [34]. Gomez et al. [19] reported that bread hardness was due to interactions between gluten and fibrous materials. Furthermore, baking conditions (temperature and time variables), state of the bread components (such as fibers, starch, gluten, whether damaged or undamaged) as well as the amount of absorbed water during dough mixing contributes to the final texture of the bread [17,19].

### 3.6. Consumer Acceptability of Magwinya

The mean scores given by consumers for appearance, taste, aroma, texture, and overall acceptability of *magwinya* samples are presented in Table 6. The mean score for overall acceptability on a nine-point Hedonic scale ranged from 5.58 to 7.58 for all five samples. The sample that did not differ significantly (*p* > 0.05) from the control in overall acceptability was the sample with 5% WB, indicating that most consumers preferred the 5% WB *magwinya* sample. Archana and Jamuna [35] in their work reported that *phulkas* and *chapathis* (unleavened bread) with a 5% WB level of incorporation, were statistically not significantly different, but at 10 and 15% level, showed extreme significant differences. However, in our work, a significant difference (*p* < 0.05) was observed between the control and composite fried dough in terms of texture, appearance, aroma, taste, and overall acceptability. These results are similar to those of Archana and Jamuna [35] who reported that the texture of *phulkas* and *chapathis*, having products with 5% WB, showed similar results with the control in this study, while products with 10 and 15% WB showed significant differences.

The overall acceptability was determined based on quality scores obtained from the evaluation of taste, texture, aroma, and appearance (crumb and crust color). At 0 and 5% WB level of substitution, *magwinya* scored highest in all four attributes, including overall acceptability, and this could be due to higher oil content of control *magwinya*, which gave it a better texture, flavor, and moist appearance. Cereal fried dough containing 20% WB received the lowest rating. Pomeranz et al. [36] reported that a progressively lesser score for the quality attributes of all bread products was observed as the level of incorporation of WB increased. For products MWB10 and MWB15, all attributes were significantly different from the control product. The appearance of *magwinya* samples in this study was rated uniform, as only fried dough with 20% WB received a lower score compared to the control. The samples fried with 20% WB differed in taste from the control, while the remaining samples had a taste comparable to the control. Evaluation of the texture also showed greater variability between sample MWB20 and the control as sample MWB20 received a significantly lower rating than the control. It is evident from the result that the control fried dough was more acceptable to the consumers, followed by *magwinya* with 5, 10, 15, and 20% level of WB supplementation. This could be attributed to the fact that consumers were becoming more accustomed to the quality attributes of the control sample. As alluded in the work of Osterman-Porcel et al. [37], the contributing attributes for consumer acceptability of the cereal fried dough containing wheat bran include taste, texture, aroma, and color. However, authors Ishida and Steel [38] in their work opined that taste and tenderness are factors to be considered in order to increase the acceptance of products containing fiber.

As further observed in the consumer acceptance scores of samples, the control cereal fried dough sample obtained a significantly high (*p* < 0.05) rating for taste when compared to other samples containing WB at a different level of substitution. The score for the taste of the control sample was also the highest among all parameters in the consumer acceptability scores of *magwinya*. In comparison to the sensory profile of samples (Table 5), the sensory attribute of flavor (sweetness) recorded high ratings, with the control sample recording a significantly high rating when compared to other cereal fried dough WB composite samples. The significantly high scores of sweetness in sensory profile and taste for consumer acceptability shows that the flavor of a product also influences the consumer acceptability of that product. This observation agrees with the findings of Serrem et al. [17], who compared the sensory characteristics and its influence on the consumer acceptability of composite biscuits by school children. 

### 3.7. Correlation between Sensory and Instrumental Measurements of Texture

Correlation between sensory and instrumental measurements of texture prompts detecting instruments to evaluate the nature of food in industries. Thus, understanding what is perceived during the sensory evaluation of the texture in the mouth and refining instrumental methods to perfect sensory evaluation, thereby anticipating the consumer’s response as the level of sympathy and overall acceptance of a new product [9,39,40]. Pearson’s correlation coefficient between sensory and instrumental texture attributes are presented in Table 7. Instrumental hardness was negatively correlated with sensory springiness (*r* = −0.63, *p* < 0.05), negatively correlated with sensory cohesiveness (*r* = −0.58, *p* < 0.05), and positively correlated with sensory chewiness (*r* = 0.30). The negative correlation between instrumental hardness and sensory springiness indicated that as the hardness of the products increased, their springiness decreased.

The significance and importance of the correlation coefficients may be relative to the type of product being studied. Instrumental cohesiveness was negatively correlated with sensory springiness (*r* = −0.71, *p* < 0.01) while sensory springiness was negatively correlated with instrumental hardness (*r* = −0.63, *p* < 0.05). The springier samples were less hard and less chewy. However, instrumental cohesiveness may not be an accurate predictor of the perceived sensory cohesiveness when comparing food samples containing different ingredients [41,42]. The description of sensory perception of cohesiveness may, thus require more than one physical measurement. The instrumental measurement of cohesiveness can provide a good evaluation of the sensory attribute of cohesiveness for some food systems such as gels, but not for other diverse food groups [43]. In this study, instrumental springiness was insignificant to either parameter due to the constant variable. Insignificant positive correlations were, however, found between sensory chewiness and all instrumental parameters. Correlation varies with the amount of mechanical deformation, sample dimensions, and type of sensory panelists (consumers or trained assessors). Further research should thus be directed towards the standardization of procedures for both sensory and instrumental testing. 

## 4. Conclusions

This work showed that the addition of WB had a significant impact on the *magwinya’s* textural, physical properties, and sensory qualities. Cereal fried dough containing more than 5% wheat bran had significantly higher hard texture than control *magwinya*, thus making them less acceptable by consumers. Composite fried dough samples containing more than 5% wheat bran were perceived as less acceptable in sensory quality, thereby revealing that panelists generally preferred *magwinya* with 5% wheat bran. However, obtained results indicate that the poor floury aroma, as well as the low specific volume of the control cereal fried dough, could be improved using wheat bran, which shows great potential as a new ingredient of fiber-enriched *magwinya*. It is advantageous to seriously explore the possibility of using wheat bran composite flours for commercial production of cereal fried/baked foods. This will result in a positive effect of reducing the cost of production, especially as wheat bran is considered a cheap waste product, thus mitigating food waste and its effects. 

## Figures and Tables

**Table 1:** Descriptive vocabulary and definitions to evaluate the sensory properties of *magwinya* samples.

Attribute	Definition	Scale anchors (0–10)
**Appearance**		
Crumb darkness	Degree of color darkness in the crust ranging from light brown to dark brown	Less dark = 1, more dark = 10
Crumb lightness	Degree of lightness in the crumb ranging from white to dark brown	Less light = 1, more light = 10
Porosity	The extent of perforation of the cereal fried dough surface and crumb. This encompasses the holes and cracks allowing the permeation of air	Less pores = 1, more pores = 10
Crust smoothness	The visual appearance of crust contour from smooth to rough	Less smooth = 1, more smooth = 10
Bran particles	The quantity of wheat bran specks on the surface	Less bran particles = 1, more bran particles = 10
Evenness of color	The evenness of color on the surface	Less evenness = 1, more evenness = 10
**Aroma**		
Yeasty	Odor associated with aromatic exchange from yeast fermentation	Less yeasty = 1, more yeasty = 10
Floury	The aromatics associated with standard baking flour, e.g., wheat flour	Less floury = 1, more floury = 10
Fermented	Intensity of aroma associated with beer	Less fermented = 1, more fermented = 10
Oily or rancid	The overall flavor impression of oil	Less oily = 1, more oily = 10
Toasted ‘burnt’	The odor impression of bread and crumb after baking/heating	Less toasted = 1, more toasted = 10
**Flavor**		
Sweet taste	Fundamental taste sensation of which sucrose is typical	Less sweet = 1, more sweet = 10
Salty taste	Fundamental taste sensation elicited by sodium chloride	Less salty = 1, more salty = 10
Oily or rancid	The overall flavor impression of oil	Less oily = 1, more oily = 10
**Oral texture**		
Hardness	Force required to bite completely through the sample placed between the molars	Less hardness = 1, more hardness = 10
Chewiness	Condition of necessitating chewing or of being difficult to chew	Less chewiness = 1, more chewiness = 10
Cohesiveness	State of cohering or sticking together	Less cohesiveness = 1, more cohesiveness = 10
Springiness	The degree or rate at which the sample returns to its original size/shape after partial compression between the tongue and palate	Less springiness = 1, more springiness = 10
**After flavor**		
Dry	Degree of which sample feels dry while chewing and absorbs saliva	Less dry = 1, more dry = 10
Lingering taste	Length of time which the taste last after the swallow	Less lingering = 1, more lingering = 10
Grittiness residue	Degree to which small particles remain	Less gritty = 1, more gritty residues = 10
Sweet aftertaste	Fundamental taste sensation of which sucrose is typical	Less sweet = 1, more sweet aftertaste = 10

**Table 2:** Color parameters of *magwinya* samples.

Color	Control	MWB5	MWB10	MWB15	MWB20
**Crumb**					
L*	62.42 ^c^ ± 1.93	56.34 ^bc^ ± 0.39	51.20 ^b^ ± 1.01	51.67 ^b^ ± 1.07	47.20 ^a^ ± 1.51
a*	1.94 ^a^ ± 0.81	5.68 ^b^ ± 0.54	7.40 ^c^ ± 0.44	7.96 ^c^ ± 0.15	8.98 ^d^ ± 0.15
b*	20.13 ^a^ ± 1.10	19.61 ^a^ ± 0.36	19.04 ^a^ ± 0.19	19.49 ^a^ ± 0.90	19.70 ^a^ ± 0.99
Chroma	20.23 ^a^ ± 1.15	20.42 ^a^ ± 0.49	20.55 ^a^ ± 0.31	21.06 ^a^ ± 0.76	21.65 ^a^ ± 0.86
Hue	84.55 ^d^ ± 2.08	73.86 ^c^ ± 1.20	68.76 ^b^ ± 0.97	67.76 ^ab^ ± 1.29	65.46 ^a^ ± 1.53
∆E	-	7.18 ^a^ ± 0.35	12.53 ^b^ ± 1.08	12.36 ^b^ ± 1.05	16.80 ^c^ ± 1.44
**Crust**					
L*	35.92 ^b^ ± 0.44	35.92 ^b^ ± 0.59	33.67 ^b^ ± 2.11	31.00 ^a^ ± 1.05	28.98 ^a^ ± 1.27
a*	17.29 ^bc^ ± 0.38	18.23 ^c^ ± 0.44	17.09 ^b^ ± 0.36	16.23 ^b^ ± 0.57	14.96 ^a^ ± 0.93
b*	24.74 ^c^ ± 1.18	24.74 ^c^ ± 0.23	18.70 ^b^ ± 0.10	18.79 ^b^ ± 1.23	16.52 ^a^ ± 1.80
Chroma	30.19 ^bc^ ± 1.18	35.03 ^c^ ± 7.49	26.00 ^b^ ± 1.68	24.83 ^b^ ± 1.29	22.29 ^a^ ± 1.95
Hue	55.03 ^b^ ± 0.73	53.62 ^b^ ± 0.91	47.57 ^a^ ± 0.55	49.16 ^a^ ± 0.98	47.77 ^a^ ± 1.41
∆E	-	1.09 ^a^ ± 0.36	6.66 ^b^ ± 0.62	7.81 ^b^ ± 1.61	11.02 ^c^ ± 2.30

Values are means ± standard deviations from triplicate determinations. Means in the same row with different superscript are significantly different (*p* < 0.05). Control = 100% WF; MWB5 = 95% WF + 5% WB; MWB10 = 90% WF + 10% WB; MWB15 = 85% WF + 15% WB; MWB20 = 80% WF + 20% WB. MWB = *magwinya* wheat bran.

**Table 3:** Weight, volume, and specific volume of *magwinya* samples.

Properties	Control	MWB5	MWB10	MWB15	MWB20
Weight	51.57 ^b^ ± 3.53	53.83 ^b^ ± 1.99	52.10 ^b^ ± 1.95	43.10 ^a^ ± 0.79	51.83 ^b^ ± 4.54
Volume	76.67 ^a^ ± 5.77	80.00 ^a^ ± 10.00	80.00 ^a^ ± 10.00	63.33 ^a^ ± 11.55	76.67 ^a^ ± 11.55
Specific volume	1.49 ^a^ ± 0.17	1.49 ^a^ ± 0.18	1.54 ^a^ ± 0.21	1.47 ^a^ ± 0.25	1.47 ^a^ ± 0.09

Values are means ± standard deviations from triplicate determinations. Means in the same row with different superscript are significantly different (*p* < 0.05). Control = 100% WF; MWB5 = 95% WF + 5% WB; MWB10 = 90% WF + 10% WB; MWB15 = 85% WF + 15% WB; MWB20 = 80% WF + 20% WB.

**Table 4:** Texture profile analysis of *magwinya.*

Parameters	Control	MWB5	MWB10	MWB15	MWB20
Hardness (g)	475.94 ^a^ ± 36.01	500.85 ^ab^ ± 45.64	562.56 ^b^ ± 38.80	649.46 ^c^ ± 42.40	667.14 ^c^ ± 52.83
Cohesiveness	0.55 ^a^ ± 0.08	0.40 ^b^ ± 0.02	0.36 ^b^ ± 0.02	0.35 ^b^ ± 0.02	0.33 ^b^ ± 0.03
Chewiness	261.70 ^a^ ± 40.44	198.63 ^b^ ± 19.87	204.11 ^b^ ± 9.87	224.85 ^ab^ ± 13.74	221.63 ^ab^ ± 14.06
Springiness	0.98 ^a^ ± 0.00	1.00 ^a^ ± 0.00	1.00 ^a^ ± 0.00	1.00 ^a^ ± 0.00	1.00 ^a^ ± 0.00

Values are means ± standard deviations from triplicate determinations. Means in the same row with different superscript are significantly different (*p* < 0.05). Control = 100% WF; MWB5 = 95% WF + 5% WB; MWB10 = 90% WF + 10% WB; MWB15 = 85% WF + 15% WB; MWB20 = 80% WF + 20% WB.

**Table 5:** Descriptive sensory profile of wheat bran *magwinya.*

Sensory Attributes	Control	MWB5	MWB10	MWB15	MWB20
**Appearance**					
Crust-brownness	6.04 ^a^ ± 0.36	5.92 ^a^ ± 0.51	6.21 ^a^ ± 0.07	6.96 ^b^ ± 0.31	8.38 ^c^ ± 0.45
Crumb-lightness	6.71 ^c^ ± 1.19	5.83 ^bc^ ± 0.92	4.92 ^b^ ± 0.52	4.42 ^ab^ ± 0.38	2.88 ^a^ ± 1.09
Bran particles	3.04 ^a^ ± 2.62	5.29 ^ab^ ± 080	6.46 ^bc^ ± 1.01	6.75 ^bc^ ± 0.76	8.33 ^c^ ± 0.38
Crust smoothness	6.42 ^d^ ± 0.47	5.79 ^cd^ ± 0.64	4.58 ^bc^ ± 1.12	4.00 ^ab^ ± 0.78	2.96 ^a^ ± 0.19
Evenness	0.40 ^a^ ± 1.23	6.33 ^a^ ± 1.08	5.67 ^a^ ± 0.45	5.92 ^a^ ± 0.95	6.29 ^a^ ± 0.52
Porosity	4.04 ^a^ ± 1.13	4.08 ^a^ ± 0.44	4.33 ^a^ ± 0.83	4.42 ^a^ ± 0.26	4.67 ^a^ ± 1.02
**Aroma**					
Yeasty	4.63 ^ab^ ± 0.75	3.63 ^a^ ± 0.82	4.21 ^ab^ ± 0.44	4.42 ^ab^ ± 0.63	5.08 ^b^ ± 0.73
Floury	4.38 ^a^ ± 0.57	4.17 ^a^ ± 0.69	4.08 ^a^ ± 0.36	4.46 ^a^ ± 0.38	4.21 ^a^ ± 0.50
Fermented	4.63 ^a^ ± 0.13	4.58 ^a^ ± 0.94	4.25 ^a^ ± 0.78	4.38 ^a^ ± 0.78	4.58 ^a^ ± 1.05
Toasted ‘burnt’	3.42 ^a^ ± 0.85	4.63 ^ab^ ± 0.65	4.92 ^b^ ± 0.73	5.13 ^b^ ± 0.76	5.75 ^b^ ± 0.75
Oily	4.71 ^a^ ± 0.69	4.21 ^a^ ± 1.21	4.58 ^a^ ± 0.47	3.96 ^a^ ± 1.34	3.33 ^a^ ± 0.31
**Flavor**					
Sweetness	5.63 ^b^ ± 0.57	5.58 ^ab^ ± 0.38	5.29 ^ab^ ± 0,26	5.00 ^ab^ ± 0.75	4.54 ^a^ ± 0.59
Saltiness	2.17 ^a^ ± 0.51	2.54 ^a^ ± 0.19	2.75 ^a^ ± 0,88	2.92 ^a^ ± 0.81	2.54 ^a^ ± 0.47
**Texture**					
Hardness	3.92 ^a^ ± 1.40	4.63 ^ab^ ± 1.02	5.54 ^abc^ ± 1.20	6.17 ^b^ ± 0.07	7.21 ^c^ ± 0.19
Cohesiveness	5.29 ^ab^ ± 1.40	5.08 ^a^ ± 1.02	5.08 ^a^ ± 1.20	5.42 ^ab^ ± 0.07	6.33 ^b^ ± 0.19
Springiness	4.17 ^a^ ± 1.28	5.08 ^ab^ ± 0.62	5.42 ^ab^ ± 0.26	5.83 ^b^ ± 0.47	6.21 ^b^ ± 1.00
Chewiness	5.54 ^a^ ± 0.59	5.46 ^a^ ± 0.14	5.38 ^a^ ± 0.50	5.42 ^a^ ± 0.26	4.79 ^a^ ± 0.19
**Aftertaste**					
Sweetness	4.83 ^a^ ± 0.07	5.04 ^a^ ± 0.63	5.13 ^a^ ± 0.33	5.29 ^a^ ± 0.79	5.17 ^a^ ± 0.51
Gritty (residues)	4.50 ^a^ ± 0.88	4.38 ^a^ ± 0.82	4.75 ^a^ ± 0.33	5.08 ^a^ ± 0.51	4.88 ^a^ ± 1.02
Lingering	4.71 ^a^ ± 0.72	4.75 ^a^ ± 1.07	4.83 ^a^ ± 0.62	5.21 ^a^ ± 0.85	5.63 ^a^ ± 0.82
Dry	4.12 ^a^ ± 1.35	4.88 ^ab^ ± 1.52	5.54 ^ab^ ± 0.26	6.08 ^bc^ ± 0.19	7.33 ^c^ ± 0.26

Values are means ± standard deviations from triplicate determinations. Means in the same row with different superscript are significantly different (*p* < 0.05). Control = 100% WF; MWB5 = 95% WF + 5% WB; MWB10 = 90% WF + 10% WB; MWB15 = 85% WF+ 15% WB; MWB20 = 80% WF + 20% WB.

**Table 6:** Consumer acceptance scores of *magwinya.*

Parameters	Control	MWB5	MWB10	MWB15	MWB20
Appearance	7.48 ^d^ ± 1.44	7.05 ^cd^ ± 1.44	6.05 ^b^ ± 1.88	6.49 ^bc^ ± 1.87	4.36 ^a^ ± 2.36
Aroma	7.18 ^c^ ± 1.51	7.00 ^c^ ± 1.50	6.39 ^b^ ± 1.38	6.45 ^b^ ± 1.53	5.09 ^a^ ± 2.31
Texture	7.58 ^c^ ± 1.52	7.18 ^c^ ± 1.61	6.46 ^b^ ± 1.41	6.60 ^b^ ± 1.78	4.78 ^a^ ± 2.41
Taste	7.71 ^c^ ± 1.32	7.35 ^bc^ ± 1.7	6.94 ^b^ ± 1.56	6.91 ^b^ ± 1.53	5.60 ^a^ ± 2.14
Overall acceptability	7.58 ^c^ ± 1.32	741 ^c^ ± 1.41	6.83 ^b^ ± 1.46	6.92 ^b^ ± 1.48	5.58 ^a^ ± 2.04

Values are means ± standard deviations from eighty consumers. Means in the same row with different superscript are significantly different (*p* < 0.05). Control = 100% WF; MWB5 = 95% WF + 5% WB; MWB10 = 90% WF + 10% WB; MWB15 = 85% WF + 15% WB; MWB20 = 80% WF + 20% WB.

**Table 7:** Correlation between the instrumental and sensory texture attributes of *magwinya.*

Sensory Texture Attributes	Instrumental Texture Attributes		
	Ihardness	Icohesiveness	Ichewiness
Hardness	−0.81 **	−0.61 *	−0.74 **
Cohesiveness	−0.58 *	−0.05	−0.32
Springiness	−0.63 *	−0.71 **	−0.67 **
Chewiness	0.30	0.35	0.30

** Correlation is significant at the 0.01 level. * Correlation is significant at the 0.05 level.
